# Glucosylceramides From *Lomentospora prolificans* Induce a Differential Production of Cytokines and Increases the Microbicidal Activity of Macrophages

**DOI:** 10.3389/fmicb.2019.00554

**Published:** 2019-03-22

**Authors:** Mariana Ingrid Dutra da Silva Xisto, Julián Esteban Muñoz Henao, Lucas dos Santos Dias, Giulia Maria Pires Santos, Renata de Oliveira Rocha Calixto, Mariana Collodetti Bernardino, Carlos Pelleschi Taborda, Eliana Barreto-Bergter

**Affiliations:** ^1^Laboratório de Química Biológica de Microrganismos, Departamento de Microbiologia Geral, Instituto de Microbiologia Paulo de Góes, Centro de Ciências da Saúde, Universidade Federal do Rio de Janeiro, Rio de Janeiro, Brazil; ^2^Studies in Translational Microbiology and Emerging Diseases Research Group, School of Medicine and Health Sciences, Universidad del Rosario, Bogotá, Colombia; ^3^Institute of Biomedical Sciences, Department of Microbiology, Medical Mycology Laboratory, Medical School and Tropical Medicine Institute, University of São Paulo, São Paulo, Brazil; ^4^Department of Pediatric, School of Medicine and Public Health, University of Wisconsin-Madison, Madison, WI, United States; ^5^Instituto Biomédico, Departamento de Microbiologia e Parasitologia, Universidade Federal Fluminense, Niterói, Brazil

**Keywords:** *Lomentospora prolificans*, glucosylceramides, GlcCer, structural characterization, innate immune response

## Abstract

*Lomentospora prolificans* is an emerging opportunistic fungus with a high resistance to antifungal agents and it can cause localized infections in immunocompetent patients and disseminated infections with a high mortality rate in immunosuppressed patients. Glucosylceramides (GlcCer) are synthetized in the majority of known fungal pathogens. They are bioactive molecules presenting different functions, such as involvement in fungal growth and morphological transitions in several fungi. The elucidation of the primary structure of the fungal surface glycoconjugates could contribute for the understanding of the mechanisms of pathogenicity. In this work, GlcCer species were isolated from mycelium and conidia forms of *L. prolificans* and their chemical structures were elucidated by mass spectrometry (ESI-MS). GlcCer purified from both forms presented a major species at *m/z* 750 that corresponds to *N*-2-hydroxyhexadecanoyl-1-β-D-glucopyranosyl-9-methyl-4,8-sphingadienine. Monoclonal antibodies against GlcCer could recognize *L. prolificans* GlcCer species from mycelium and conidia, suggesting a conserved epitope in fungal GlcCer. In addition, *in vivo* assays showed that purified GlcCer species from both forms was able to induce a high secretion of pro-inflammatory cytokines by splenocytes. GlcCer species also promote the recruitment of polymorphonuclear, eosinophils, small peritoneal macrophage (SPM) and mononuclear cells to the peritoneal cavity. GlcCer species were also able to induce the oxidative burst by peritoneal macrophages with NO and superoxide radicals production, and to increase the killing of *L. prolificans* conidia by peritoneal macrophages. These results indicate that GlcCer species from *L. prolificans* are a potent immune response activator.

## Introduction

*Lomentospora prolificans* (formerly *Scedosporium prolificans*) (Lackner et al., 2014) is a human pathogen presenting high virulence and antifungal multidrug resistance, being able to cause infections in immunocompetent and immunocompromised patients (Cortez et al., 2008; Lackner and Guarro, 2013). The cell wall glycoconjugates from *Pseudallescheria*/*Scedosporium* complex have been studied and these molecules are essential for the virulence and other biological activities (Pinto et al., 2002; Rollin-Pinheiro et al., 2014). Glucosylceramides are the main neutral glycosphingolipids synthetized in the majority of known fungal pathogens (Barreto-Bergter et al., 2004; Pinto et al., 2008). GlcCer is associated with fungal growth and morphological transitions in *Cryptococcus neoformans*, *P. boydii, Candida albicans, Aspergillus fumigatus*, and *Colletotrichum gloeosporioides* (Rodrigues et al., 2000; Barreto-Bergter et al., 2004, 2011; da Silva et al., 2004; Nimrichter et al., 2005; Rollin-Pinheiro et al., 2016).

Anti-GlcCer mAb protects mice against lethal *C. neoformans* infection (Rodrigues et al., 2000). *In vitro* synergistic interactions were observed between the mAb against GlcCer and both amphotericin B and itraconazole suggesting the combined use of monoclonal antibodies against GlcCer and antifungal drugs for antifungal immunotherapy (Rollin-Pinheiro et al., 2014).

GlcCer structures similar to those previously described for *S. apiospermum*, *S. aurantiacum* and *P. minutispora* have been isolated in several fungi from the *Pseudallescheria*/*Scedosporium* complex (Calixto et al., 2016). Thus, elucidation of the primary structure of fungal GlcCer that function as virulence determinant is important for understanding the mechanism of fungal pathogenicity. In this study, we report the characterization of GlcCer species in *L. prolificans* and the involvement of these molecules in the activation of the innate immune response.

## Materials and Methods

### Microorganism and Growth Conditions

A culture of *Lomentospora prolificans* (strain FMR3569), was supplied by Dr. J. Guarro, Unitat de Microbiologia, Facultat de Medicina e Institut d’Estudis Avançats, Réus, Spain. It was grown in Erlenmeyer flasks containing 200 ml of Sabouraud modified medium, and incubated at room temperature for 7 days with shaking (pre-inoculum). Cultures were then transferred to the same medium and incubated for 7 days with shaking. The mycelium was filtered, washed with distilled water, and stored at −20°C. Conidia were grown on Petri plates containing Sabouraud modified medium at room temperature. After 7 days, conidia were obtained by washing the plate surface with phosphate-buffered saline (PBS) and hyphal fragments and debris were removed by filtration through gauze.

### Extraction and Purification of GlcCer From *L. prolificans*

Intact hyphae and conidia of *L. prolificans* were successively extracted at room temperature using chloroform:methanol at 2:1 and 1:2 (v/v) ratios. The extracts were combined and dried, and the crude lipid extract was partitioned as described by Folch et al. (1957). The lipids recovered from the Folch lower layer were fractionated on a silica gel column and eluted sequentially with chloroform, acetone and methanol. The acetone and methanol fractions containing glycosphingolipids were then further purified according to Rollin-Pinheiro et al. (2014).

### Sugar Analysis

In order to analyze the monosaccharide components, GlcCer hydrolysis was performed with 3 M trifluoroacetic acid at 100°C for 3 h and the monosaccharides were identified by HPTLC using sugar standards, according to Rollin-Pinheiro et al. (2014).

### ESI-MS Analysis of *L. prolificans* GlcCer Species

Mass spectrometry was carried out by electrospray ionization (ESI-MS) in positive mode according to Calixto et al. (2016).

### Reactivity of Anti-GlcCer mAb With Purified GlcCer Species and Swollen Conidia

The reactivity of *L. prolificans* GlcCer species to anti-GlcCer mAb was analyzed by ELISA according to Nimrichter et al. (2005). Briefly, *L. prolificans* GlcCer species from mycelia and conidia were dissolved in ethanol:methanol 1:1 (v/v), and 1 μg/well was added to a flat-bottomed polystyrene microtiter plate (BD-Falcon, Sparks, MD, United States). The plate was dried and blocked with PBS containing 1% BSA (2 h, 37°C). Decreasing concentrations of the anti-GlcCer mAb and an unrelated IgG were added, and the plate was incubated at 37°C for 1 h. The plate was washed three times and then incubated with HRP-conjugated anti-mouse IgG (1:1,000 dilution) (Sigma-Aldrich, Germany) for 1 h at 37°C. The plate was washed again three times with PBS and the antigen antibody complexes were detected with 0.04% ortho-phenylenediamine (OPD) in phosphate-citrate buffer at pH 5.0 containing 30 vol. H_2_O_2_ and the absorbance was measured at 490 nm using spectrophotometer (Bio-Rad, United States).

Using 4% paraformaldehyde-fixed swollen conidia as antigens, 1 × 10^6^ cells were added to a flat-bottomed polystyrene microtiter plate (BD-Falcon, Sparks, MD, United States) in 50 μl PBS, followed by incubation for 1 h at 37°C and then overnight at 4°C. Plates were washed three times with washing buffer (10 mM PBS-buffered saline, 0.1% Tween 20 [pH 7.2]) and blocked with 1% BSA in PBS (blocking buffer). Anti-GlcCer mAb and an unrelated IgG were used in different concentrations (100–3.13 μg/ml) as primary antibody and incubated at 37°C for 1 h (Lopes et al., 2010). Rabbit anti-*L. prolificans* serum and rabbit pre-immune serum were added as a positive and negative control, respectively. After this incubation, the procedures were made as described above.

### Immunostaining of *L. prolificans* GlcCer Species

GlcCer species from mycelia and conidia of *L. prolificans* were submitted to immunostaining as described by Rodrigues et al. (2000).

### Immunofluorescence Analysis

Mycelium and conidia were fixed with 4% paraformaldehyde in 0.1 M cacodylate buffer (pH 7.0) for 1 h. The cells were adhered on slides coated with poly-L-lysine for 10 min. A solution of 3% BSA in 0.1 M cacodylate buffer pH 7.0 was used as blocking agent for 1 h. Primary antibody (anti-GlcCer mAb) was added to the system at a concentration of 50 μg/ml and incubated at 4°C overnight in a moist chamber. Secondary antibodies conjugated with Alexa Fluor 546 were incubated under the same conditions. After three washes, the slides were observed in an Olympus AX70 fluorescence microscope (Olympus America Inc., Center Valley, PA, United States) using a 620-nm filter and a100× magnification lens. Some conidia were treated with 1M NaOH under agitation overnight to do a partial depletion of melanin in *L. prolificans* cells. Moreover, some other conidia were incubated in RPMI medium for 6 h to allow germination before being set.

### Mice

In all experiments female BALB/c mice, 4–8 weeks old, were used (Lopes et al., 2010). All experiments were performed according to Institutional Animal Welfare Committee of the Universidade de São Paulo (USP), Protocol 101/2014/CEUA-USP.

### Phagocytosis and Killing of *L. prolificans*

Mice received intraperitoneal (i.p.) injection of 200 μg of GlcCer species with 0.5% DMSO in PBS or only 0.5% DMSO in PBS as a control, both solutions were filtered in 0.22 μm filter before i.p. injection. Three days after injection, macrophages were collected from the PerC with RPMI-1640 ice-cold. The phagocytes were centrifuged (10 min at 1,350 rpm), counted in Neubauer’s chamber, and plated in RPMI1640 supplemented with 10% heat inactivated fetal bovine serum. For the phagocytosis and killing process, conidia from *L. prolificans* were added in the macrophages monolayer at the *L. prolificans*/macrophage ratio of 5:1 and incubated for 2 h. After this incubation, macrophages were gently washed with RPMI medium to remove non-internalized conidia. The phagocytic index (PIs) was calculated according to Bittencourt et al. (2006).

The killing of *L. prolificans* by macrophages was assessed in the same monolayers. After removal of the non-phagocytized *L. prolificans* conidia, macrophages were lysed with sterile cold water. To quantify the viable intracellular *L. prolificans*, the resulting suspension was spread on Sabouraud agar plates and incubated at 30°C for 48 h. The growth was determined by comparing the number of colonies forming units (CFU) of *L. prolificans* conidia incubated with macrophages stimulated with GlcCer species or 0.5% DMSO in PBS as a control.

### Quantification of Nitric Oxide and Hydrogen Peroxide Released by Macrophages

Peritoneal macrophages were plated at 2 × 10^5^ cells/well in 96-well polystyrene tissue-culture plates. Heat-killed conidia (ratio 5:1), GlcCer species from conidial and mycelial forms (50 and 100 μg/ml), or LPS (O111:B4 – 10 ng/well) were incubated with macrophages for 2 and 24 h at 37°C in 5% CO_2_. After 2 and 24 h of incubation, the supernatants were collected. Nitric oxide quantification was carried out after 24 h of incubation using a commercial Griess reagent kit (Promega) (Xisto et al., 2015). Similarly, reactive oxygen intermediates were measured after 2 h of incubation MTT method according to Lopes et al. (2010).

### Determination of Cytokine Levels in the Spleen

BALB/c mice were stimulated by i.p. via with 200 μg of GlcCer species in 0.5% DMSO or only 0.5% DMSO as a control, both solutions were filtered in 0.22 μm filter before i.p. injection. After 24 h the spleen of each animal was extracted, weighed, macerated in protease inhibitor (Complete EDTA-free Roche) and centrifuged. The supernatant from each sample was collected and stored at 80°C until cytokine assays were performed. The cytokines TNF-α, IFN-γ, IL-17, and IL-10, and the chemokine MIP-2/CXCL2 were evaluated in the supernatant of the spleens. Cytokines were quantified according to the manufacturer (Duo Set ELISA Kit R&D Systems; BD OptEIA ELISA Set). Polymyxin B (10 μg/ml) was added to the GlcCer species before the immunoassays.

### *Ex vivo* Cytokine Assay

Mice received i.p. injection of 200 μg of GlcCer species in 0.5% DMSO in PBS or only 0.5% DMSO in PBS as a control, both solutions were filtered in 0.22 μm filter before i.p. injection. After 3 days, mice were sacrificed and the peritoneal macrophages were plated in 96-well plates (2.5 × 10^5^). Adherent cells were stimulated for 18 h in RPMI-1640 medium with GlcCer species (100 μg/ml) or LPS (O111:B4 – 10 ng/well), after that time the supernatant was collected, centrifuged (12,000 rpm for 10 min) for removal of cellular debris and stored at −80°C. The concentration of TNF-α and IL-10 was determined by ELISA according to the manufacturer’s instructions (BD OptEIA, Set ELISA mouse). Polymyxin B (10 μg/ml) was added 5 min before the addition of the stimulus, to rule out the possibility that the stimulating activity was due to contaminating lipopolysaccharides.

### Recruitment of Cells to the Peritoneum Cavity

Peritoneal cells were harvested 24 h after injection of 0.5% DMSO in PBS (vehicle) or GlcCer species from mycelium (200 μg). Both solutions were filtered in 0.22 μm filter before i.p. injection. The PerC was washed with 10 ml of cold RPMI-1640 and then the cells were counted in Neubauer chamber. Peritoneal cells (2 × 10^6^) were incubated at 4°C for 30 min with the following fluorochrome conjugated mAb in an 11-color staining combination: PE-labeled anti-CD45 (clone 30-F11; Biolegend, United States); BV785-labeled anti-CD19 (clone 6D5; Biolegend, United States); PerCP-Cy5.5-labeled anti-CD4 (clone RM4-5; BD, United States); BV650-labeled anti-CD8 (clone 53-6.8; Biolegend, United States); PE-Cy7-labeled anti-CD11b (clone M1/70; BD, United States); APC-Cy7-labeled anti-CD11c (clone N418; Biolegend, United States); Alexa Fluor 700-labeled anti-Ly6G (clone 1A8; Biolegend, United States); BV605-labeled anti-Ly6C (clone AL21; BD, United States); APC-labeled anti-MHC II (clone M5/114.15.2; eBioscience, United States). After washing to remove unbound antibodies, the peritoneal cells were incubated at room temperature for 30 min with a commercial viability stain (LIVE/DEAD Fixable Violet stain, V500; Life Technologies, United States). The cells were washed, fixed with 1% paraformaldehyde and analyzed on cytometer LSR Fortesa (BD, United States). A total of 100,000 events was acquired and the data was analyzed with FlowJo software (TreeStar Inc., United States). We used the strategy of fluorescence-minus-one (FMO) stains sets to distinguish autofluorescent cells from cells with low levels of the marker of interest (Supplementary Figure S1). Polymyxin B (10 μg/ml) was added to the GlcCer species before the immunoassays.

### Statistical Analysis

Data were presented as mean values ± SD and were compared using one-way ANOVA test with Tukey multiple comparisons post-test. The unpaired Student’s *t*-test with Welch’s correction (two-tailed) was used for comparison of two groups when the data met the assumption of *t*-tests. GraphPad Prism 5.0 software was used to carried out the analysis. *P*-values were considered significant when *p* < 0.05.

## Results

### Glucosylceramide Purification and Structural Analysis

Crude lipid extraction and GlcCer species purification were carried out as previously described (Rollin-Pinheiro et al., 2014; Calixto et al., 2016). After extraction with mixtures of chloroform and methanol followed by different steps of chromatographic separation, orcinol-reactive bands were detected by HPTLC.

**FIGURE 1:**
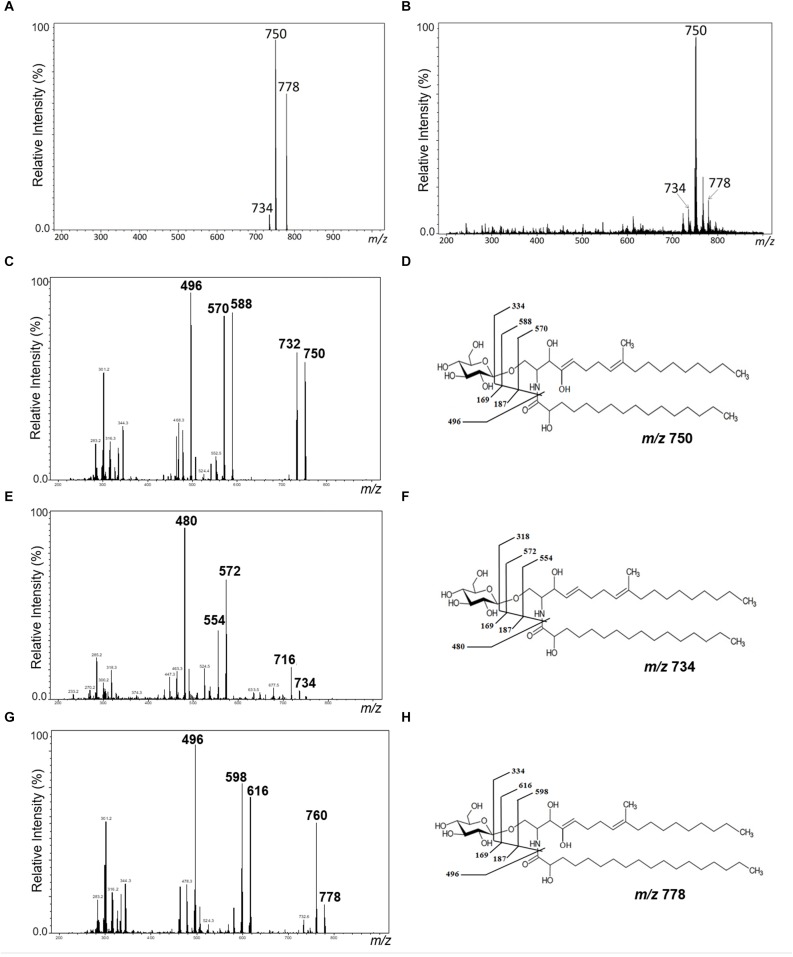
Positive ESI-MS [M+Li] analysis of GlcCer species from *L. prolificans*. ESI-MS1 of GlcCer species from mycelial **(A)** and conidial **(B)** forms. ESI-MS2 of the ions species *m/z* 750, *m/z* 734, and *m/z* 778 **(C**,**E,G)** and proposed structures for each GlcCer species **(D**,**F,H)** from mycelial form.

Purified GlcCer species from conidia and mycelium of *L. prolificans* were analyzed by positive ion mode ESI-MS. GlcCer purified from both mycelia and conidia are composed of three molecular species. A major species at mass to charge ratio (*m/z*) 750 [M+Li]+, while two minor ion species were observed at *m/z* 734 and *m/z* 778 (Figure 1A,B).

**FIGURE 2:**
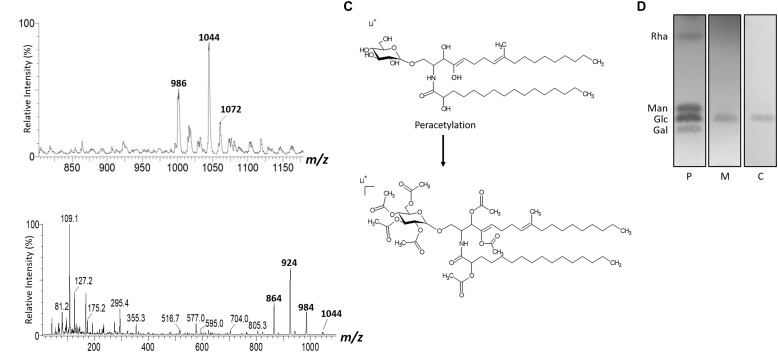
ESI-MS1 **(A)** and MS2 **(B)** of the peracetylated GlcCer species (*m/z* 1044) from mycelium form. The assignment proposed for native (*m/z* 750) and peracetylated GlcCer species (*m/z* 1044) from *L. prolificans* **(C)**. HPTLC of the monosaccharide constituent of GlcCer **(D)**.

The GlcCer molecular species were analyzed by ESI-MS/MS (Figure 1C–H). The loss of 162 units, common to all GlcCer analyzed and diagnostic of a monosaccharide unit, gave rise to daughter ions at *m/z* 572, *m/z* 588 and *m/z* 616. [M-hexose+Li+] corresponding to the ceramide monolithiated ion from the parental ions at *m/z* 734, 750 and 778, respectively. Other prominent fragments at *m/z* 732, 716 and 760 corresponded to loss of water from [M+Li]+. The daughter ions at *m/z* 480 and *m/z* 496 observed are consistent with a loss of hydroxylated C16 fatty acid. The difference of 16 units observed suggests that the long chain base present in the major species (*m/z* 750) could possibly present an extra hydroxyl group. This hypothesis was strongly supported after analysis of the peracetylated GlcCer (*m/z* 750) derivatives showing a molecular and monolithiated ion at *m/z* 1044, consistent with an addition of seven acetyl group units (Figure 2A–C). Monolithiated ions at *m/z* 986 and *m/z* 1072 corresponded to peracetylated GlcCer species (*m/z* 734 and *m/z* 778) derivatives, respectively (Figure 2A). Other mass signals were present at *m/z* 984 [M+Li – HO-Ac], *m/z* 924 [M+Li – 2 HO-Ac] and m/z 864 [M+Li – 3HO-Ac], in the MS2 spectrum of the peracetylated GlcCer (*m/z* 750) (Figure 2B). Glucose was the only monosaccharide identified by HPTLC and was present in three species of GlcCer analyzed (Figure 2D).

### mAb Binding to GlcCer Species and Its Distribution on the Surface of *L. prolificans*

**FIGURE 3:**
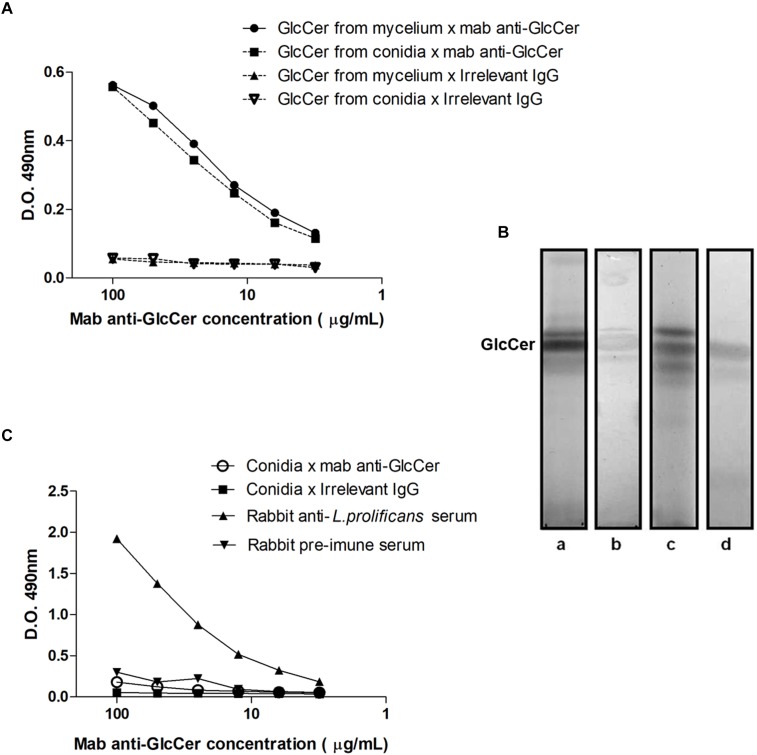
Reactivity anti-GlcCer mAb with GlcCer species from *L. prolificans*. **(A)** ELISA of mAb binding to GlcCer species from mycelial and conidial forms of *L. prolificans*. Irrelevant IgG antibody was used as a negative control. **(B)** Immunostaining of GlcCer species from mycelial and conidial forms of *L. prolificans*. HPTLC was developed with CHCl3:MeOH:NH_4_OH 2 M (40:10:1 v/v). (Columns **a** and **c**) Orcinol/H_2_SO_4_ positive fractions for the mycelium and conidium, respectively; and (columns **b** and **d)** Reactivity of the anti-GlcCer mAb with the GlcCer species from mycelium and conidium forms, respectively. **(C)** Binding of anti-GlcCer mAb to the conidia of *L. prolificans*. Rabbit anti-*L. prolificans* serum was used as a positive control. The ELISA was performed in triplicate.

**FIGURE 4:**
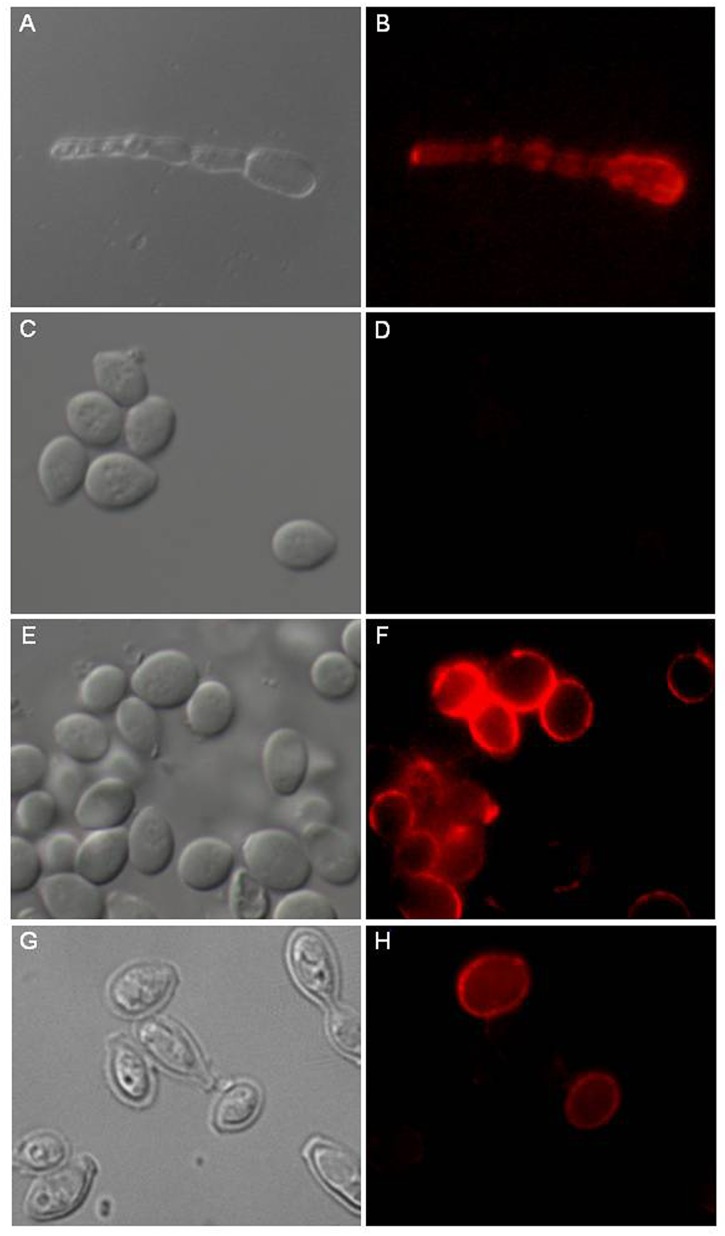
Distribution of GlcCer species on *L. prolificans* surface. The anti-GlcCer mAb revealed the presence of GlcCer species on the mycelium surface **(B)**. The anti-GlcCer mAb was not able to bind to the surface of the intact conidium **(D)**. After incubation in RPMI medium for 6 h as a swollen conidium **(F)** or treatment with 1M NaOH **(H)**. GlcCer species were identified on the conidial surface with the anti-GlcCer mAb. In **(A**,**C**,**E,G)** was differential interferencial contrast microscopy (DIC). Bar: 10 μm.

We observed that *L. prolificans* GlcCer isolated either from mycelium or conidia are similarly recognized by anti-GlcCer antibodies (Figure 3A). In addition, HPTLC immunostaining revealed that anti-GlcCer antibody recognized bands co-migrating with conidia and mycelium *L. prolificans* GlcCer (Figure 3B). However, the indirect ELISA assay showed that the anti-GlcCer mAb was not able to bind to the surface of conidia, suggesting that the GlcCer is not exposed on the surface of this form (Figure 3C). On the other hand, mycelial forms of *L. prolificans* were reactive with this antibody (Figure 4).

Therefore, we evaluated that the absence of GlcCer immunodetection in conidia cells could be a consequence of GlcCer masking by an external layer of other fungal components that makes the GlcCer inaccessible to antibodies.

Based on the alkali solubility of *L. prolificans* melanin, the relationship between melanin expression and GlcCer recognition was then investigated. Alkali-treated conidia were strongly recognized by the monoclonal antibody to GlcCer, indicating that an alkali soluble component, possibly melanin, was hindering the antibody access to the inner GlcCer (Figure 4G,H).

Moreover, conidia were incubated for 6 h at 37°C in RPMI medium to initiate the germination process by forming swollen conidia. These swollen conidia were fixed and incubated with monoclonal antibody to GlcCer. mAb was able to recognize GlcCer on the surface of swollen conidia (Figure 4E,F).

### Involvement of GlcCer Species on Nitric Oxide and Superoxide Release by Macrophages

**FIGURE 5:**
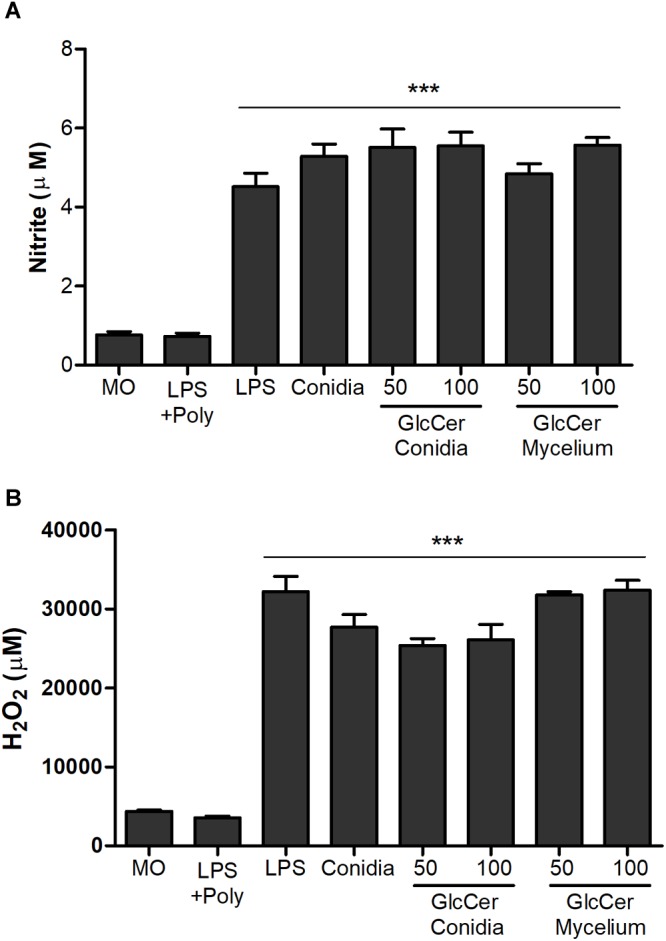
Activation of oxidative burst from peritoneal macrophages. 2.5 × 10^5^ macrophages were incubated in the presence of 50 and 100 μg/ml at 37°C, for 2 and 24 h for quantification of NO **(A)** and H_2_O_2_ **(B)**, respectively. As positive controls, LPS (10 ng/well) or *L. prolificans* conidia (5:1) were used. Data are the mean ± SEM of duplicate samples from four independent experiments. ^∗∗∗^*p* < 0.0001; +Poly, polymyxin added.

To detect the involvement of conidia and mycelial GlcCer in the immune response, we measured the capacity of these molecules to induce NO and superoxide by peritoneal macrophages. We observed that the macrophages stimulated with conidia or mycelial GlcCer had a significant increase in the NO amounts compared to non-stimulated macrophages at the tested concentrations (Figure 5A). Macrophages also produced superoxide radicals in response to the stimulation with GlcCer (Figure 5B). It is interesting to note that the effect of GlcCer in the production of NO and superoxide is very similar to the one obtained when a potent stimulus, like LPS, is used (Figure 5B).

### GlcCer Species Increase theMicrobicidal Function of Macrophages

**FIGURE 6:**
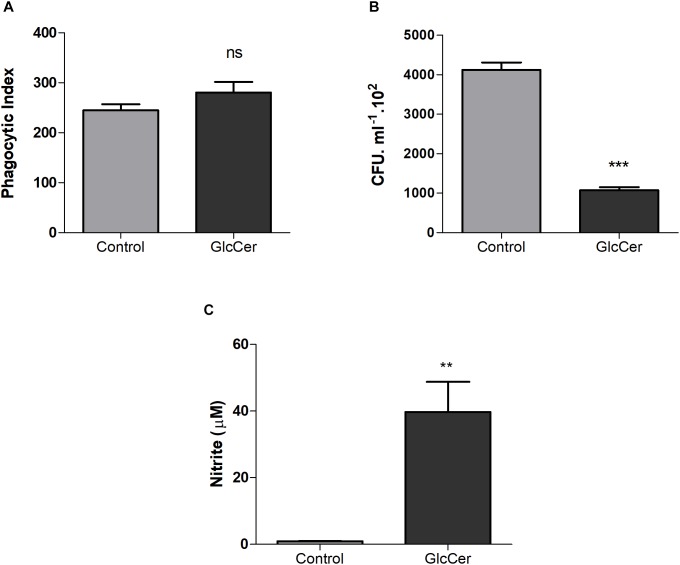
Microbicidal activity of macrophages. **(A)** Phagocytosis of the *L. prolificans* conidia by macrophages previously activated or not by GlcCer species *in vivo* (200 μg/100 μl i.p.). **(B)** Microbicidal capacity of macrophages previously activated or not by GlcCer species *in vivo* (200 μg/100 μl i.p.) and incubated for 2 h with conidia of *L. prolificans*. After this incubation the macrophages were lysed and the supernatant was plated on solid Sabouraud. The CFU count was performed after 48 h. **(C)** NO production by macrophages previously activated or not by GlcCer species *in vivo* (200 μg/100 μl i.p.). Data are the mean ± SEM of duplicate samples from four independent experiments. ^∗∗^*p* < 0.001; ^∗∗∗^*p* < 0.0001; ns, no significant.

Phagocytosis and killing of *L. prolificans* conidia by peritoneal macrophages were evaluated after the stimulation of mice with GlcCer species (200 μg/mouse, i.p.) or only 0.5% DMSO in PBS as a control. After 3 days, mice were sacrificed and the peritoneal macrophages were plated. The stimulation with GlcCer was not able to increase conidial phagocytosis, as compared to the control (Figure 6A). However, the macrophages microbicide capacity increased since the activation of macrophages *in vivo* with GlcCer led to death of more than 50% of the internalized conidia in relation to the control (Figure 6B). In addition, it was possible to observe that macrophages stimulated *in vivo*, and plated later, were able to produce high concentrations of NO (Figure 6C), suggesting that previous activation of macrophages led to an increased microbicide capacity due to the increase of NO production.

### GlcCer Species Induce the Production of TNF-α in Macrophages

**FIGURE 7:**
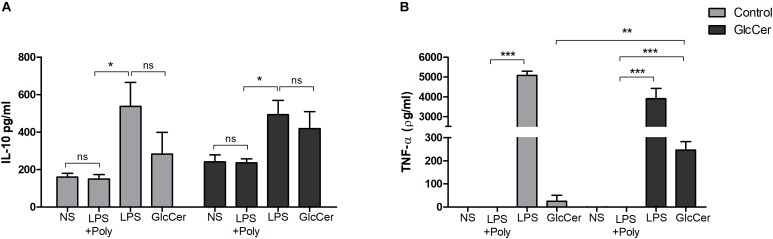
Induction of cytokine secretion by peritoneal macrophages. Mice were stimulated with 200 μg/100 μl i.p. and 72 h later the macrophages were obtained from the peritoneal lavage and plated. In the control group the animals were stimulated with 0.5% DMSO in PBS. After incubation with the GlcCer species for 18 h, the culture supernatant was collected and the concentration of IL-10 **(A)** and TNF-α **(B)** were determined by ELISA. ^∗^*P* < 0.05; ^∗∗^*p* < 0.001; ^∗∗∗^*p* < 0.0001; ns, no significant; NS, no stimulus; +Poly, polymyxin added.

The ability of GlcCer to stimulate cytokine secretion was evaluated in macrophages previously activated or not with GlcCer species (200 μg/ml) *in vivo*. The peritoneal macrophages were then plated, and incubated with GlcCer species (100 μg/ml) for 18 h. LPS was used as a positive control (10 ng/well). The culture supernatant was collected and the concentration of TNF-α and IL-10 cytokines was assessed. GlcCer species were only able to induce TNF-α secretion in macrophages previously stimulated with GlcCer *in vivo.* GlcCer species were not able to induce IL-10 secretion even if it was previously stimulated with GlcCer species (Figure 7).

### GlcCer Species Induce the Production of Pro-inflammatory Cytokines *in vivo*

**FIGURE 8:**
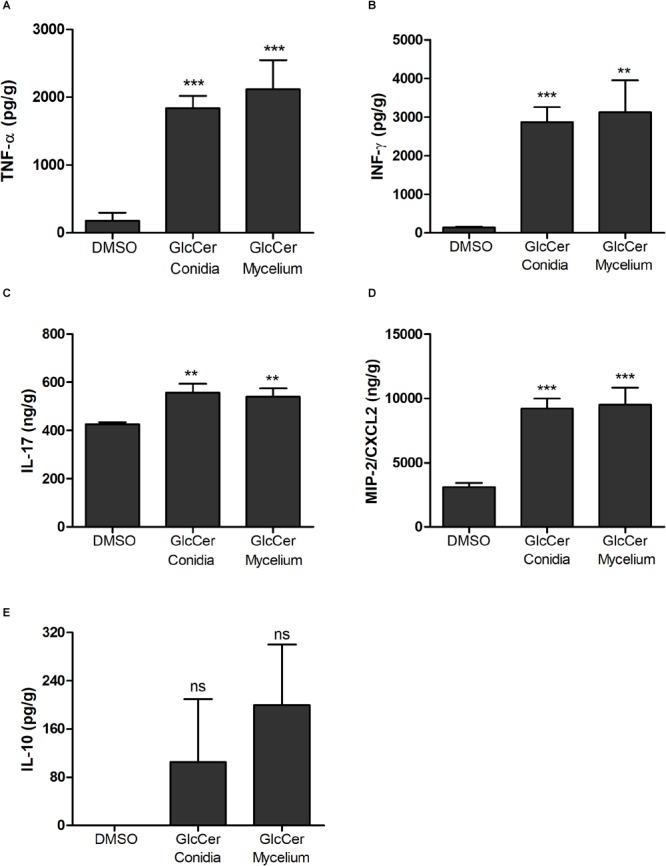
Cytokine detection in spleens from mice 24 h after GlcCer species injection. The pro-inflammatory cytokines analyzed were: **(A)** TNF-α; **(B)** INF-γ; **(C)** IL-17; **(D)** MIP2/CXCL2; and anti-inflammatory cytokine **(E)** IL-10. Each group was injected with 200 μg of mycelium or conidia form, and as a control 0.5% of DMSO in PBS. ^∗∗^*p* < 0.005; ^∗∗∗^*p* < 0.0001; ns, no significant.

Cytokine production by spleen was evaluated after exposition with GlcCer species. Our results demonstrate that GlcCer species from both mycelium and conidia stimulated the secretion of TNF-α, IFN-γ, IL-17 and the chemokine MIP-2/CXCL2 (Figure 8A–D). In contrast to the results observed for pro-inflammatory cytokines, production of IL-10 was not consistent (Figure 8E).

### GlcCer Species Induce Leukocyte Recruitment *in vivo*

**FIGURE 9:**
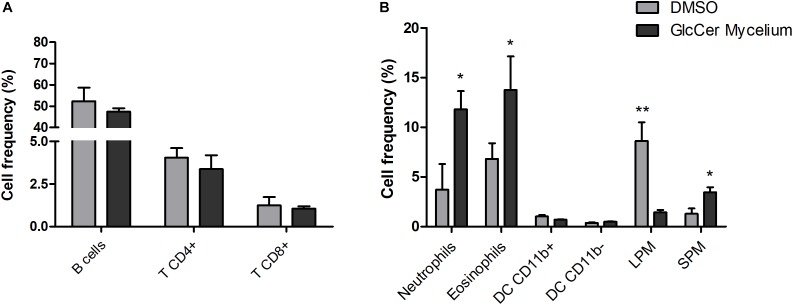
GlcCer species from *L. prolificans* mycelium induce recruitment of neutrophils, eosinophils and small peritoneal macrophage (SPM) in the peritoneal cavity 24 h after the injection. Mice were treated intraperitoneally with 0.5% DMSO in PBS (DMSO) or 200 μg GlcCer species from mycelium, and after 24 h lymphoid **(A)** and myeloid cells **(B)** in the peritoneal cavity were evaluated by flow cytometry. DC, dendritic cell; LPM, large peritoneal macrophage; SPM, small peritoneal macrophage. The results were analyzed with paired *t*-test with ^∗^*p* ≤ 0.02 or ^∗∗^*p* ≤ 0.005.

Since GlcCer is able to induce macrophage activation as observed for the NO and H_2_O_2_ production, we investigated a possible pro-inflammatory activity of GlcCer species *in vivo* in an experimental model of peritonitis induced by the challenge with purified GlcCer species. 24 h after the injection GlcCer species (200 μg), lymphoid and myeloid cells in the PerC were evaluated by flow cytometry. The lymphoid cells analyzed were B, TCD4^+^ and TCD8^+^ cells and myeloid cells were neutrophils, eosinophils and subpopulations of peritoneal macrophages (SPM and LPM). Our results showed that GlcCer species were able to promote the recruitment of neutrophils, eosinophils and SPM when compared to animals treated with 0.5% DMSO in PBS (vehicle), indicating a pro-inflammatory activity of the GlcCer species (Figure 9B). GlcCer species were not able to promote the recruitment of lymphoid and LPM cells (Figure 9A).

## Discussion

Glucosylceramides are the main neutral glycosphingolipids expressed in fungal pathogens. They have different functions, such as the involvement in fungal growth and morphological transitions in *C. neoformans*, *C. albicans*, *A. fumigatus*, *C. gloeosporioides*, and *P. boydii* and *S. apiospermum*, belonging to the *Pseudallescheria/Scedosporium* complex (Rodrigues et al., 2000; Barreto-Bergter et al., 2004, 2011; da Silva et al., 2004; Nimrichter et al., 2005).

In this work, GlcCer species obtained from the mycelial and conidial forms of *L. prolificans* had their chemical structures elucidated by mass spectrometry (ESI-MS). The ability of GlcCer species to activate a pro-inflammatory immune response with the increased microbicidal effect of macrophages was also studied.

The GlcCer structures found in both forms of *L. prolificans* were similar and showed a predominant molecular species with *m/z* 750, corresponding to the structure *N*-2-hydroxyhexade canoic-1-*β*-D-glucopyranosyl-9-methyl-4,8-sphingadienine. An additional hydroxyl group is present in the long chain base component of ceramide. Two other species with *m/z* 734 and *m/z* 778, which differ in the absence of the additional hydroxyl group and the fatty acid chain length were also present. After an acid hydrolysis of the GlcCer species of both forms and a subsequent analysis by high resolution thin layer chromatography using different monosaccharide standards, it was possible to identify that the hexose present in the GlcCer species of *L. prolificans* is glucose. Similar structures have been described in *S. apiospermum* with molecular ion of *m/z* 734. Treatment of the fungal conidia mAbs against GlcCer, caused a significant inhibition of the cellular growth. On the other hand, this effect was not observed in the mycelia of *S. apiospermum* due to the GlcCer localization that avoid the mAb recognition (Rollin-Pinheiro et al., 2014). *F. pedrosoi* also presents the GlcCer with molecular ions of *m/z* 750. However, the mAb recognition depends of the fungal dimorphism and the melanization process (Nimrichter et al., 2005).

Purified human antibodies against GlcCer obtained from sera of patients with cryptococcosis, recognized the cell wall of the fungus showing a decrease in growth and multiplication of *Cryptococcus neoformans.* A similar result was found in sera from patients with paracoccidioidomycosis, aspergillosis and histoplasmosis (Rodrigues et al., 2000).

The reactivity of anti-CMH mAb, obtained using a GlcCer isolated from *A. fumigatus* (Rollin-Pinheiro et al., 2014) with the GlcCer species of both forms of *L. prolificans* was evaluated by ELISA and immunostaining. The anti-CMH mAb was able to react with the purified GlcCer species of *L. prolificans* thus evidencing the structural similarity of these molecules, presenting common epitopes recognized by this antibody, and confirming once again that the GlcCer is a conserved structure present in fungal cells (Rollin-Pinheiro et al., 2016). Despite being able to react with GlcCer species isolated from mycelium and conidia forms, anti-CMH mAb did not react with resting conidia of *L. prolificans* by ELISA. Immunofluorescence experiments using the anti-CMH mAb has been shown that GlcCer species are accessible in the mycelium form, but not in the conidia form. Similar result was observed in conidia of *P. boydii* (Pinto et al., 2002). Previous work from our group showed that melanin present in the cell wall of the dematiaceous fungus *Fonsecaea pedrosoi*, could block the GlcCer recognition. Alkali-treated cells, became efficiently recognized by the anti-CMH antibody (Nimrichter et al., 2005).

*L. prolificans* is able to produce the polymer dihydroxynaphthalene (DHN)-melanin via a biosynthetic pathway (Revankar and Sutton, 2010; Al-Laaeiby et al., 2016). *L. prolificans* resting conidia were then treated with 1M NaOH overnight at room temperature for partial removal of melanin and subsequently incubated with the anti-CMH mAb, which it then recognized the GlcCer species on the surface of the conidia. Moreover, the anti-CMH mAb was able to recognize GlcCer species on the surface of swollen conidia. Our results suggest that GlcCer species are masked on the surface of young conidia due to the presence of alkali-soluble pigments, and during the germination process the GlcCer species begin to stay accessible on the surface of conidia to hyphae.

Elimination of invasive pathogens by macrophages occurs by the release of reactive intermediates of oxygen and nitrogen (Roilides et al., 2009). The oxidative response plays a key role in host resistance to fungal infections, although several fungi have an antioxidant system that determines their adaptation and resistance in the host (Hamilton and Holdom, 1999; Lima et al., 2007). In this sense, we evaluated the production of superoxide radicals by the quantification of H_2_O_2_, and NO in peritoneal macrophages stimulated with the GlcCer species from mycelial and conidium forms. Both GlcCer species of *L. prolificans* were able to induce the release of superoxide radicals and NO by macrophages, with the same intensity as the positive controls, LPS and conidia, demonstrating their participation in the activation of innate immunity processes.

Several studies using *in vitro* models suggest that the activation of macrophages and the consequent production of microbicidal compounds, including NO, depends on signals mediated by cytokines, such as INF-γ and TNF-α, and PAMPS such as LPS (Green et al., 1990; Amber et al., 1991). In other study *Histoplasma capsulatum* naive macrophages are permissive to the growth of *H. capsulatum*, while cytokine-activated macrophages are able to block the multiplication of this fungus (Brummer and Stevens, 1995). Macrophages previously primed with GlcCer species *in vivo* did not increase the phagocytosis of the conidia of *L. prolificans*, compared to the control, but the fungus recovery, quantified by the CFU count, was significantly lower than the control, demonstrating that the GlcCer species were able to activate the macrophages leading to the elimination of more than 50% of the internalized conidia. In addition, it was also observed that macrophages previously primed with GlcCer species *in vivo* produced a significantly increase in NO compare with control, suggesting that previous activation of macrophages with GlcCer species contributes to the increase of its microbicidal capacity. Moreover, we evaluated the secretion of cytokines TNF-α and IL-10 by these macrophages previously activated or not with GlcCer species *in vivo*. It was possible to observe that non-activated macrophages *in vivo* with GlcCer species were not able to carry TNF-α or IL-10 secretion, but macrophages activated *in vivo* with GlcCer species were able to secrete the pro-inflammatory cytokine TNF-α but not IL-10. Our results suggest that GlcCer species are not able to induce the secretion of TNF-α directly, but indirectly through another cell of the immunity, activating the macrophage. GlcCer species would then serve as the second signal and induce secretion of TNF-α by peritoneal macrophages.

GlcCer species from mycelium or conidia was able to induce a high secretion of the pro-inflammatory cytokines TNF-α, INF-γ, IL-17 and the chemokine MIP-2/CXCL-2, but it was not able to stimulate the secretion of the anti-inflammatory cytokine IL-10. These results suggesting that GlcCer species are potent activators of pro-inflammatory cytokine secretion which is crucial in the differentiation of T CD4 lymphocytes fungal-specific in Th1 and Th17 which are crucial for defense against fungal infections (Espinosa and Rivera, 2012).

Since GlcCer species of *L. prolificans* were able to stimulate the chemokine secretion in the spleen, we investigated the profile of cells recruited by GlcCer species in the PerC. We observed that GlcCer species from mycelium was able to recruit polymorphonuclear, eosinophils, and SPM and mononuclear cells to the PerC, and consequently the activation of the pro-inflammatory response. The recruitment of SPM and mononuclear cells to the PerC induced by CMH show the same behavior as previously demonstrated in the literature (Cain et al., 2013). In this work, reduction of LPM, increase of SPM, short live cells originated from the circulating monocytes and recruitment of inflammatory monocytes cells in the PerC were observed. These cells seem to contribute to the effector function of PerC producing high levels of NO, IL-12, MIP-1α, TNF-α, and RANTES (Ghosn et al., 2010; Cassado Ados et al., 2011; Cain et al., 2013).

In summary, the results obtained in this work indicate that GlcCer species from *L. prolificans* are potent immune response activators. These molecules induce a strong production of NO in peritoneal macrophages with a high killing activity. *In vivo*, GlcCer species induce an immune response composed by a Th1 and Th17 cytokine profile, with recruitment of inflammatory cells to the PerC. In this way, we believed that these molecules are very important to the immune response against *L. prolificans*, and could be used to produce antibodies, vaccines or as an adjuvant.

## Data Availability

All datasets generated for this study are included in the manuscript and/or the Supplementary Files.

## Ethics Statement

This study was carried out in accordance with the recommendations of Institutional Animal Welfare Committee of the Universidade de São Paulo (USP). The protocol was approved by the 101/2014/CEUA-USP.

## Author Contributions

MX, JH, LD, and EB-B designed the experiments and drafted the manuscript. MX, JH, LD, GS, RC, and MB performed all the experiments. MX, JH, LD, CT, and EB-B analyzed the data. CT critically revised the manuscript. All authors read and approved the manuscript.

## Conflict of Interest Statement

The authors declare that the research was conducted in the absence of any commercial or financial relationships that could be construed as a potential conflict of interest.

## Supplementary Material

FIGURE S1 Flow Cytometry analysis of the cells recruited to the peritoneum cavity. Mice were injected intraperitoneally with 0.5% DMSO in PBS (vehicle) or 200 μg of GlcCer from mycelium. After 24 h, the mice were euthanized and the cells recruited to peritoneal cavity was evaluated by flow cytometry. The panel with 10 colors was designed to distinguished some types of cell: CD4 T, CD8 T, B cells, neutrophils, eosinophils, large peritoneal macrophage (LPM), small peritoneal macrophage (SPM), CD11c+CD11b+MHCII+ and CD11c+ CD11b-MHC+. All the gates were sat up based on the FMO controls.

## Abbreviations

ESI-MS: electrospray ionization-mass spectrometry
GlcCer: glucosylceramide
LPM: large peritoneal macrophage
PerC: peritoneal cavity
SPM: small peritoneal macrophage
TLC: thin-layer chromatography.
