# Supplementary figures and images for: Glucosylceramides From Lomentospora prolificans Induce a Differential Production of Cytokines and Increases the Microbicidal Activity of Macrophages
Source: Front Microbiol. 2019 Mar 22;10:554. doi: 10.3389/fmicb.2019.00554 (PMC6440385; doi:10.3389/fmicb.2019.00554)

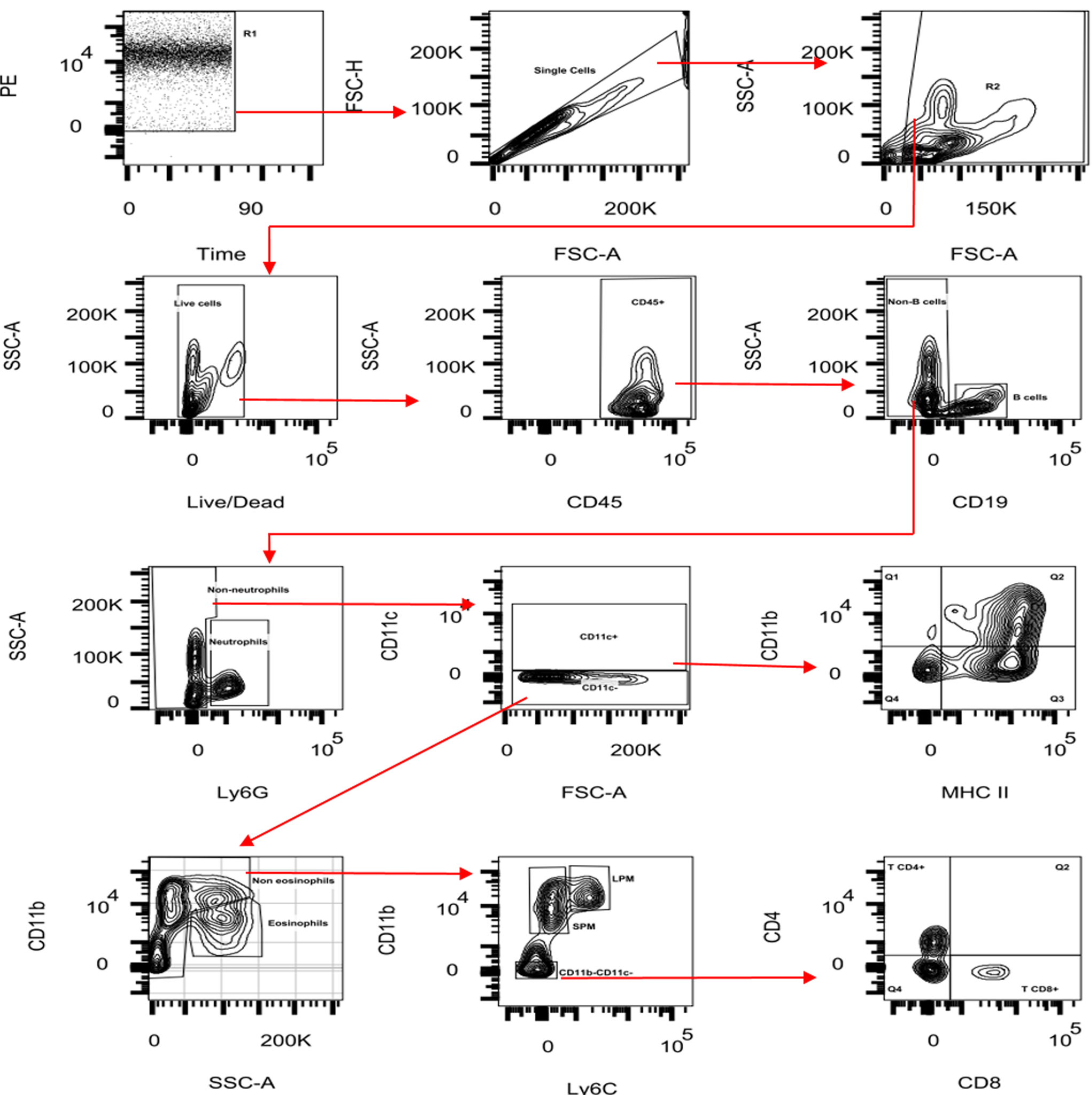

Supplement: FIGURE S1 — Flow Cytometry analysis of the cells recruited to the peritoneum cavity. Mice were injected intraperitoneally with 0.5% DMSO in PBS (vehicle) or 200 μg of GlcCer from mycelium. After 24 h, the mice were euthanized and the cells recruited to peritoneal cavity was evaluated by flow cytometry. The panel with 10 colors was designed to distinguished some types of cell: CD4 T, CD8 T, B cells, neutrophils, eosinophils, large peritoneal macrophage (LPM), small peritoneal macrophage (SPM), CD11c+CD11b+MHCII+ and CD11c+ CD11b-MHC+. All the gates were sat up based on the FMO controls. [file Image_1.TIF]
